# Lectures on Medical Pathology

**Published:** 1886-12

**Authors:** 


*Lectures on Medical Pathology.* By H. GAWEN SUTTON, F.R.C.P. Pp. 215. London: BALLIÈRE & Co. 1886.

These lectures were, we are told in the Preface, delivered spontaneously; and they are published from shorthand notes taken during their delivery.

The lectures have all the freedom, crispness and vigour of such spontaneous effusions. They are exceedingly readable, full of suggestive thoughts, utterly devoid of system, and, in expression as well as in matter, very original. They stimulate thought, rather than exhaust knowledge.

One delightful element in these lectures is their brevity. As they are shorthand reproductions, they are no doubt full, and one envies the student who has to listen to a lecture which occupies only three small pages of print. But some of them occupy from ten to nineteen pages; fortunately these are few.

Another pleasing feature is the rhetorical display occasionally indulged in by the author. Scarcely a figure of speech is unrepresented, and not a few poets are quoted. Some of his sentences are efforts of genius. "Health, then, is going 'easy' in everything," he tells us. No healthy man will worry over bills and taxes and patients, we conclude.

Some of the descriptions are graphic. Thus: "It is a terrible thing for a poor patient to be coughing up blood. One of the bravest men I knew, a captain in the army, had profuse hæmoptysis, and I had to quiet his fears. I was anxious to get him home alive. I did that, and helped him by saying, 'Cough it up, captain!' Fear tends to paralyse breathing, and paralyse the right side of the heart:—what chance has a poor fellow if he is coughing up blood and is taken with fear?" What indeed! Not many people cough up blood with perfect *sang froid;* and when they do not, it is well to know what words to use by way of encouragement, as well as what drugs to prescribe.

Pathological processes are represented in dashing little miniatures, which remind one forcibly of the works produced by a certain class of artists, known as "Impressionists." The representation is broad, suggestive, and facile; full of life, and dealing with only such details as are essential to the conception. There is no elaboration; only clear, broad, distinct touches. In this view we commend the work to our readers' notice.

